# How Long Can Stool Samples Be Fixed for an Accurate Diagnosis of Soil-Transmitted Helminth Infection Using Mini-FLOTAC?

**DOI:** 10.1371/journal.pntd.0003698

**Published:** 2015-04-07

**Authors:** Beatrice Barda, Marco Albonico, Davide Ianniello, Shaali M. Ame, Jennifer Keiser, Benjamin Speich, Laura Rinaldi, Giuseppe Cringoli, Roberto Burioni, Antonio Montresor, Jürg Utzinger

**Affiliations:** 1 Department of Medical Parasitology and Infection Biology, Swiss Tropical and Public Health Institute, Basel, Switzerland; 2 University of Basel, Basel, Switzerland; 3 Ivo de Carneri Foundation, Milano, Italy; 4 Section of Veterinary Parasitology and Parasitic Diseases, University of Naples Federico II, Naples, Italy; 5 Laboratory Division, Public Health Laboratory–Ivo de Carneri, Chake-Chake, Tanzania; 6 Laboratory of Microbiology and Virology, San Raffaele Hospital, Milan, Italy; 7 Department of Control of Neglected Tropical Diseases, World Health Organization, Geneva, Switzerland; 8 Department of Epidemiology and Public Health, Swiss Tropical and Public Health Institute, Basel, Switzerland; Universidade Federal de Minas Gerais, BRAZIL

## Abstract

**Background:**

Kato-Katz is a widely used method for the diagnosis of soil-transmitted helminth infection. Fecal samples cannot be preserved, and hence, should be processed on the day of collection and examined under a microscope within 60 min of slide preparation. Mini-FLOTAC is a technique that allows examining fixed fecal samples. We assessed the performance of Mini-FLOTAC using formalin-fixed stool samples compared to Kato-Katz and determined the dynamics of prevalence and intensity estimates of soil-transmitted helminth infection over a 31-day time period.

**Methodology:**

The study was carried out in late 2013 on Pemba Island, Tanzania. Forty-one children were enrolled and stool samples were subjected on the day of collection to a single Kato-Katz thick smear and Mini-FLOTAC examination; 12 aliquots of stool were fixed in 5% formalin and subsequently examined by Mini-FLOTAC up to 31 days after collection.

**Principal Findings:**

The combined results from Kato-Katz and Mini-FLOTAC revealed that 100% of children were positive for *Trichuris trichiura*, 85% for *Ascaris lumbricoides*, and 54% for hookworm. Kato-Katz and Mini-FLOTAC techniques found similar prevalence estimates for *A*. *lumbricoides* (85% *versus* 76%), *T*. *trichiura* (98% *versus* 100%), and hookworm (42% *versus* 51%). The mean eggs per gram of stool (EPG) according to Kato-Katz and Mini-FLOTAC was 12,075 and 11,679 for *A*. *lumbricoides*, 1,074 and 1,592 for *T*. *trichiura*, and 255 and 220 for hookworm, respectively. The mean EPG from day 1 to 31 of fixation was stable for *A*. *lumbricoides* and *T*. *trichiura*, but gradually declined for hookworm, starting at day 15.

**Conclusions/Significance:**

The findings of our study suggest that for a qualitative diagnosis of soil-transmitted helminth infection, stool samples can be fixed in 5% formalin for at least 30 days. However, for an accurate quantitative diagnosis of hookworm, we suggest a limit of 15 days of preservation. Our results have direct implication for integrating soil-transmitted helminthiasis into transmission assessment surveys for lymphatic filariasis.

## Author Summary

Soil-transmitted helminths are parasitic worms (hookworm, roundworm, and whipworm) that affect hundreds of millions of people. Kato-Katz is the most widely used technique for the diagnosis of soil-transmitted helminth infection. It requires the collection, processing, and microscopic examination of stool samples within the same day. In remote areas, laboratories are often far away from where stool samples are being collected, which makes it difficult to examine the samples on the same day. Mini-FLOTAC is an alternative to Kato-Katz technique that allows analysis of fixed stool samples several days after collection. We assessed the accuracy of the Mini-FLOTAC with fixed stool samples. The study was carried out in late 2013 on Pemba Island and 41 children participated. Fresh stool samples were first examined by Kato-Katz and then fixed in formalin and examined by Mini-FLOTAC (12 examinations within one month). We found that for a qualitative diagnosis (presence or absence of infection) stool samples can be fixed for 31 days. However, for an accurate quantitative diagnosis of hookworm eggs, stool samples should not be stored for more than 15 days, as egg counts decline. Our results have implications for integrating soil-transmitted helminth surveys with other neglected tropical diseases.

## Introduction

Kato-Katz technique is the standard method recommended by the World Health Organization (WHO) for the diagnosis of intestinal schistosomiasis and soil-transmitted helminthiasis [1,2]. By counting helminth eggs in a given amount of stool, this method allows not only determining the presence of infection, but also its intensity, as expressed in eggs per gram of feces (EPG) [3,4]. Kato-Katz technique requires processing fecal specimens preferentially within 24 hours from production and collection in the field, in order to minimize degradation of hookworm eggs [1,4–6]. This strict time requirement entails that, during field surveys, the team collecting fecal samples either performs the microscopic examination on the spot or transfers the samples to a nearby laboratory for work-up the same day [7].

FLOTAC technique [8] has been developed in veterinary parasitology for the diagnosis of intestinal parasites, and it has been adopted in human parasitology due to its high sensitivity [8–10]. However, FLOTAC technique is more time-consuming than Kato-Katz method and requires some specific laboratory equipment (e.g., large bucket centrifuge with special adaptors) [11]. Recently, FLOTAC has been simplified and Mini-FLOTAC has been developed in order to meet the needs of resource-limited settings. Indeed, Mini-FLOTAC is simple to apply, it allows the analysis of fixed fecal samples, and helminth eggs can be quantified, as with Kato-Katz technique [12].

The possibility of collecting fecal specimens in the field, adding a fixative, and analyzing the samples several days later in a central laboratory could overcome the time limitation of working on fresh samples, and hence improve the easiness and the quality of soil-transmitted helminthiasis diagnosis. For example, it has been suggested to integrate soil-transmitted helminthiasis within transmission assessment surveys (TAS) that are conducted in the context of the program to eliminate lymphatic filariasis [13–15]. Usually, the TAS team remains in a school (or a village) only for few hours for collecting and analyzing blood samples [16]. While this short time frame would allow collection and fixing of stool samples with formalin for subsequent soil-transmitted helminth diagnosis in the laboratory, it would not suffice to prepare and microscopically examine Kato-Katz thick smears on the spot. Hence, the use of a method that does not limit the time-to-process the stool specimens in the laboratory holds promise to be included in TAS in areas where soil-transmitted helminthiasis and lymphatic filariasis are co-endemic. Additionally, Mini-FLOTAC is a closed system, and the safe handling, together with fixing of stool samples, protect the operator from potential contamination [12, 17]. However, the length of storage time of fixed fecal samples to maintain reliable and accurate diagnostic performance of the Mini-FLOTAC for the detection and quantification of soil-transmitted helminth eggs has not been evaluated before.

The aim of this study was to assess the accuracy of the Mini-FLOTAC technique on fecal samples which had been fixed for up to 31 days (from stool collection to microscope examination) in maintaining good/optimal correlation in terms of prevalence and intensity of infections and a good/robust “microscope readability” (the ease by which the different soil-transmitted helminth eggs can be identified over time).

## Methods

### Ethics Statement

This study was embedded in a randomized controlled trial to assess the efficacy and safety of different anthelmintic drugs against *Trichuris trichiura* and concurrent soil-transmitted helminth infections [18]. In brief, the study was approved by the ethics committees of Basel, Switzerland (EKBB; reference no. 123/13) and the Ministry of Health and Social Welfare of Zanzibar, United Republic of Tanzania (ZAMREC; reference no. 0001/June/13). The trial is registered at controlled-trials.com (identifier: ISRCTN80245406). Written informed consent was obtained from the parents/guardians of the children before enrolment. Data were anonymized and confidentiality assured throughout the study. Data files were stored in a safe cabinet within the Public Health Laboratory-Ivo de Carneri (PHL-IdC) and children were identified by code. At the end of the study, all children were treated with a single oral dose of albendazole (400 mg) as part of the mass drug administration intervention of the national neglected tropical diseases (NTD) control program, implemented in January 2014.

### Study Site

The study was carried out on Pemba Island, United Republic of Tanzania, in October and November 2013. Pemba is part of the Zanzibar archipelago, together with the main island of Unguja and located few degrees south of the Equator, about 50 km off the coast of mainland Tanzania. Pemba is an island where soil-transmitted helminth infections are still widespread despite deworming activities that have been implemented over the past 20 years [19]. Indeed, numerous epidemiological surveys and clinical trials have been conducted, which unanimously report a high prevalence and intensity of soil-transmitted helminth infection [19–23].

### Study Population and Sample Selection

Embedded in a clinical trial which assessed the efficacy and safety of different drug combinations against *T*. *trichiura* and concomitant soil-transmitted helminth infections [18], 41 children from the primary schools of Mchamgandogo and Shungi were selected for our study. The inclusion criteria were (i) double or triple infection with *T*. *trichiura*, *A*. *lumbricoides*, and/or hookworm in order to obtain at least 20 infections for each of the three soil-transmitted helminth species; and (ii) no recent (within the last 6 months) anthelmintic treatment. In view of the second criterion, we chose those children who had initially been screened for soil-transmitted helminth infection in September 2013, but were not subjected to treatment in any of the trial arms and had not yet been treated by the national NTD control program (done only after the clinical study in November 2013, namely in January 2014).

### Parasitology

We collected one fecal sample of about 50 g from each child. Samples were transferred to the PHL-IdC. On the same day (D_1_), each sample was divided into 12 Fill-FLOTAC (reusable plastic containers used to collect, homogenize, and filter stool samples). Each Fill-FLOTAC contained 2 g of stool, weighted with a digital scale (CS 200 Compact Scale; People’s Republic of China, precision 0.1 g), and 2 ml of 5% formalin (dilution 1:1). Each Fill-FLOTAC was used to perform Mini-FLOTAC at different time intervals post stool collection and fixing until day 31 (D_31_).

On the collection day (D_1_), each sample was subjected to a single Kato-Katz thick smear and to a single Mini-FLOTAC. Briefly, Kato-Katz thick smear was performed using a 41.7 mg template following a standard protocol and fecal egg counts were multiplied by a factor 24 to obtain an estimate of EPG [2, 24]. Mini-FLOTAC technique was performed using saturated saline as flotation solution (FS no. 2), with a sample dilution ratio of 1:20; Mini-FLOTAC chambers were filled with 1 ml of sample solution per chamber [12]. The fecal egg count of each sample was multiplied by a factor 10 to obtain an estimate of EPG, therefore the sensitivity detection limit of Mini-FLOTAC was 10 EPG. From D_1_ until day 15 (D_15_) the samples were analyzed every other day, and from day 16 (D_16_) until day 31 (D_31_) the samples were analyzed every third day with a single Mini-FLOTAC. All Fill-FLOTAC containers were stored at room temperature (between 20 and 30°C) at PHL-IdC throughout the study.

Quality control was carried out on 10% of Kato-Katz [25] and Mini-FLOTAC slides that have been re-checked by skilled microscopists with experience on both methods. The classification of light, moderate, and heavy infection was done according to WHO recommendations: for *A*. *lumbricoides*: light 1–4,999 EPG, moderate 5,000–49,999 EPG, and heavy infection ≥50,000 EPG; for *T*. *trichiura*: light 1–999 EPG, moderate 1,000–9,999 EPG, and heavy infection ≥10,000; for hookworm: light 1–1,999 EPG, moderate 2,000–3,999 EPG, and heavy infection ≥4,000 EPG [26].

In order to assess the diagnostic accuracy over time, we evaluated the shape and contrast from the background of the different soil-transmitted helminth eggs by taking sample photographs of the different eggs during the study, randomly chosen among the samples. Moreover, readability of helminth eggs on the slides was assessed by asking laboratory technicians about the ease in recognizing the different eggs under the microscope at each time point on all samples.

### Statistical Analysis

Data were entered into an Excel file. Analysis was performed using SPSS 16.0 EV (WinWrap Basic, 1993–2007). The results were analyzed by 2x2 contingency tables. Pearson index was calculated to assess the accuracy of the two diagnostic methods and their agreement. The strength of agreement criteria were: ≤0 indicating poor, 0.01–0.20 indicating slight, 0.21–0.40 indicating fair, 0.41–0.60 indicating moderate, 0.61–0.80 indicating substantial, and 0.81–1.00 indicating almost perfect agreement [27]. The comparison between arithmetic mean EPGs was calculate with Student’s *t* test for paired samples; the level of significance was set at p value <0.05, and 95% confidence intervals (CIs) were calculated.

We used an estimated ‘gold’ standard that considered any positive sample for each soil-transmitted helminth infection detected by any method at any time of examination. Accuracy and repeatability of the method was calculated throughout the 31-day period of stool preservation.

## Results

### Performance of Mini-FLOTAC and Kato-Katz Methods

The mean age of the 41 children was 11 years (range: 8–14 years); one third (n = 14) were girls. At D_1_, the number of positive samples according to Kato-Katz and the initial Mini-FLOTAC reading was 41/41 (100%) for *T*. *trichiura*, 36/41 (88%) for *A*. *lumbricoides*, and 22/41 (54%) for hookworm. The prevalence and intensity of infections according to our estimated ‘gold’ standard (a single Kato-Katz thick smear plus multiple Mini-FLOTAC) are shown in Tables 1 and 2.

**Table 1:** Prevalence and intensity (mean eggs per gram of stool, EPG) of soil-transmitted helminth infections at D_1_ with Kato-Katz and Mini-FLOTAC.

Helminth species	Kato-Katz thick smear	Mini-FLOTAC method	‘Gold’ standard
	n (%)	n (%)	n (%)
*Ascaris lumbricoides*	35(85)	31(76)	40 (98)
*Trichuris trichiura*	40(98)	41(100)	41 (100)
Hookworm	17(42)	21(51)	32 (78)
	EPG	EPG	
*Ascaris lumbricoides*	12074	11678	
*Trichuris trichiura*	1074	1591	
Hookworm	254	219	

“Gold” standard: combined positive resulted from Kato-Katz and Mini-FLOTAC methods

**Table 2:** Class of intensity of infections at D_1_ with Kato-Katz and Mini-FLOTAC.

	Negative		Light infection		Moderate infection		Heavy infection	
Helminth	KK n (%)	MF n (%)	KK n (%)	MF n (%)	KK n (%)	MF n (%)	KK n (%)	MF n (%)
*Ascaris lumbricoides*	6 (14.6)	10 (24.3)	15 (36.6)	9 (22)	20 (48.8)	20 (48.8)	0 (0)	2 (4.9)
*Trichuris trichiura*	1 (2.4)	0 (0)	23 (56.1)	22 (53.4)	17 (41.5)	19 (46.3)	0 (0)	0 (0)
Hookworm	24 (58.5)	20 (48.8)	16 (39)	21 (51.2)	1 (2.4)	0 (0)	0 (0)	0 (0)

KK: Kato-Katz, MF: mini-FLOTAC

Kato-Katz was more sensitive than Mini-FLOTAC for the diagnosis of *A*. *lumbricoides* (88% *vs*. 78%) at D_1_ examination. Conversely, Mini-FLOTAC showed higher sensitivity for *T*. *trichiura* (100% *vs*. 98%) and hookworm diagnosis (66% *vs*. 53%) than Kato-Katz. Table 3 shows the sensitivity of Mini-FLOTAC for detection of species-specific soil-transmitted helminth diagnosis over the 31 days of stool preservation. The sensitivity was statistically different only for hookworm for all detections except for D_3_ and D_5_ compared to our estimated ‘gold’ standard.

**Table 3:** Sensitivity of the techniques from D1 until D31.

TECHNIQUE SENSITIVITY % (95%CI)													
	KK D_1_ (95%CI)	MF D_1_ (95%CI)	MF D_3_ (95%CI)	MF D_5_ (95%CI)	MF D_7_ (95%CI)	MF D_9_ (95%CI)	MF D_11_ (95%CI)	MF D_13_ (95%CI)	MF D_15_ (95%CI)	MF D_19_ (95%CI)	MF D_23_ (95%CI)	MF D_27_ (95%CI)	MF D_31_ (95%CI)
*Ascaris lumbricoides*	87.5 (72.4–95.3)	77.5 (61.1–88.6)	77.5 (61.1–88.6)	82.5 (66.6–92.1)	85 (69.5–93.8)	77.5 (61.1–88.6)	80 (63.9–90.4)	77.5 (61.1–88.6)	80 (63.9–90.4)	77.5 (61.1–88.6)	80 (63.9–90.4)	77.5 (61.1–88.6)	80 (63.9–90.4)
*Trichuris trichiura*	97.56 (85.6–99.9)	100 (89.3–100.0)	100 (89.3–100.0)	100 (89.3–100.0)	100 (89.3–100.0)	100 (89.3–100.0)	100 (89.3–100.0)	100 (89.3–100.0)	100 (89.3–100.0)	100 (89.3–100.0)	100 (89.3–100.0)	100 (89.3–100.0)	97.56 (85.6–99.9)
Hookworm	53.1* (35.0–70.5)	65.6* (46.8–80.8)	84.3 (66.5–94.1)	78.1 (59.6–90.1)	65.6* (46.8–80.8)	68.7* (49.9–83.3)	62.5* (43.8–78.3)	62.5* (43.8–78.3)	68.75* (49.9–83.3)	62.5* (43.8–78.3)	71.9* (53.0–85.6)	65.6* (46.8–80.8)	65.6* (46.8–80.8)

* Significantly different from the ‘gold’ standard; p < 0.05

KK: Kato-Katz, MF: Mini-FLOTAC; D: day

We found a substantial agreement between the two methods for the diagnosis of *A*. *lumbricoides* (κ = 0.78) and hookworm (κ = 0.65), and a perfect agreement for the diagnosis of *T*. *trichiura* (κ = 1.0). The accordance between EPG was substantial for *A*. *lumbricoides* (κ = 0.81) and hookworm (κ = 0.73), and moderate for *T*. *trichiura* (κ = 0.45). The arithmetic mean fecal egg counts using Kato-Katz was 12,075 EPG for *A*. *lumbricoides*, 1,074 EPG for *T*. *trichiura*, and 255 EPG for hookworm. Mini-FLOTAC revealed arithmetic mean fecal egg counts of 11,679 EPG for *A*. *lumbricoides*, 1,592 EPG for *T*. *trichiura*, and 220 EPG for hookworm. There was no statistically significant difference between the mean fecal egg counts of any soil-transmitted helminth infection detected by either Kato-Katz or Mini-FLOTAC at D_1_. Similar proportions of light, moderate, and heavy infections were detected by the two techniques for *A*. *lumbricoides* (light n = 15 using Kato-Katz, and n = 9 using Mini-FLOTAC; moderate, n = 20 using either Kato-Katz or Mini-FLOTAC; heavy, n = 0 using Kato-Katz and n = 2 using Mini-FLOTAC), *T*. *trichiura* (light n = 23 using Kato-Katz, and n = 22 using Mini-FLOTAC; moderate n = 17 using Kato-Katz, and n = 19 using Mini-FLOTAC; heavy n = 0 using either Kato-Katz or Mini-FLOTAC), and hookworm (light n = 17 using Kato-Katz, and n = 21 using Mini-FLOTAC; moderate and heavy n = 0 using either Kato-Katz or Mini-FLOTAC).

### Influence of Formalin Fixation of Stool Samples for Soil-Transmitted Helminth Eggs Using the Mini-FLOTAC Technique

**Fig 1:**
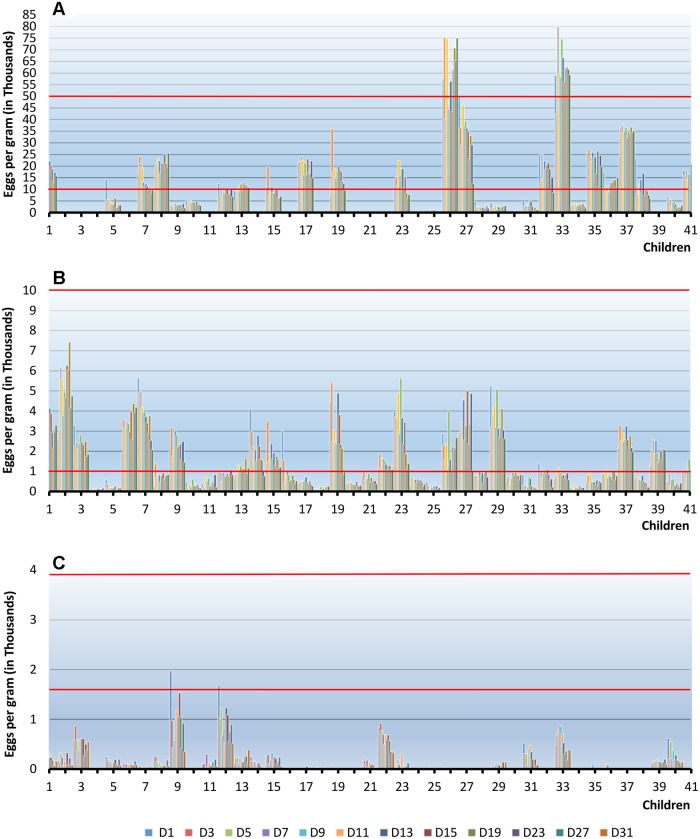
Prevalence of *A*. *lumbricoides*, *T*. *trichiura*, and hookworm over time with Mini-FLOTAC technique. KK D: Kato-Katz day 1, MF: Mini-FLOTAC.

The mean prevalence from D_1_ until D_31_ was 77.4% for *A*. *lumbricoides*, 99.8% for *T*. *trichiura*, and 53.5% for hookworm. The trend of infection prevalence is shown in Fig 1.

The agreement among the soil-transmitted helminth species-specific infection prevalence estimates over time was stable for the three soil-transmitted helminths. For *A*. *lumbricoides*, Pearson index was above 0.6 for all detections up to D_19_ and then 0.5 from D_23_ to D_31_. For *T*. *trichiura* the Pearson index was 1 throughout the study, while it was above 0.7 for hookworm, apart from detection at D_7_ that was 0.6 compared with D_3_.

**Fig 2:**
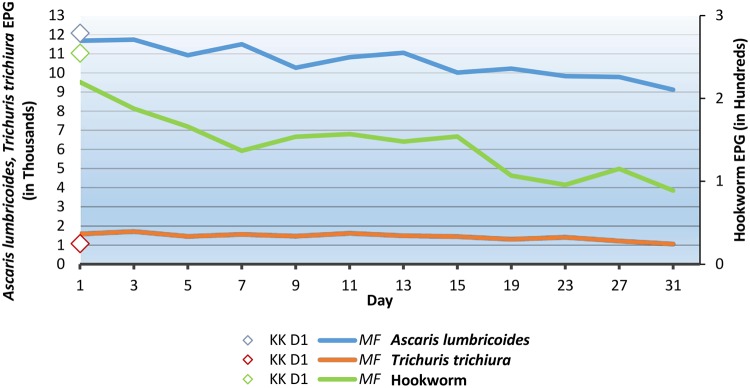
Intensity of soil-transmitted helminth infections calculated in EPG over time with the Kato-Katz and Mini-FLOTAC techniques. KK D1: Kato-Katz day 1, MF: Mini-FLOTAC.

**Fig 3:**
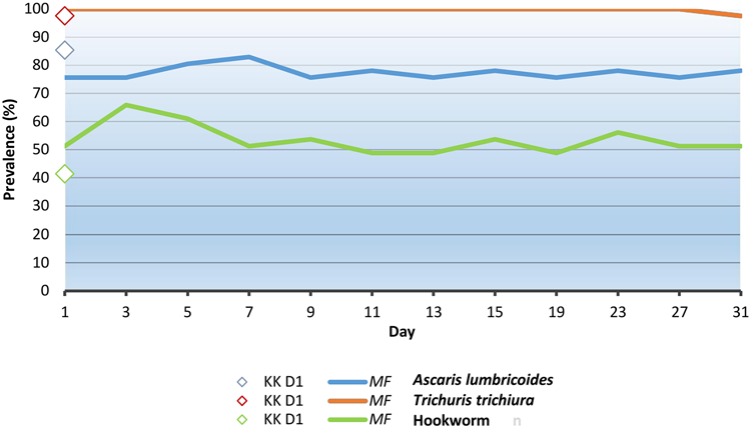
Class of soil-transmitted helminth infection intensity over time for each child using the Mini-FLOTAC technique. Red lines: class of infection: *A*. *lumbricoides*:< 5000 light, 5000–50000 moderate, >50000 heavy; *T*. *trichiura*: < 1000 light, 1000–10000 moderate, >10000 heavy; Hookworm:< 2000 light, 2000–4000 moderate, >4000 heavy.

The mean intensity of fecal egg counts from D_1_ until D_31_ was 10,582 EPG for *A*. *lumbricoides*, 1,448 EPG for *T*. *trichiura*, and 144 EPG for hookworm. The mean intensity of infection according to samples analyzed from D_1_ to D_31_ is shown in Fig 2. The calculated agreement by Pearson index between fecal egg counts was almost always above 0.9 apart from a couple of detections (0.8 between D_1_ and D_31_ and between D_3_ and D_5_) for *A*. *lumbricoides*, above 0.8 until D_13_ and 0.7 from D_15_ for *T*. *trichiura*, and above 0.8 for hookworm apart from two detections (0.7 between D_1_ and D_31_ and between D_5_ and D_19_). The major change in the estimated prevalence was associated with low intensity infections, as shown in Fig 3.

From D_1_ until D_31_ Mini-FLOTAC detections for hookworm were statistically different compared to our estimated ‘gold’ standard, except for D_3_ and D_5_, but not significantly different among each other. The Student’s *t* test showed no significant difference among the mean fecal egg counts of any soil-transmitted helminth infection from D_1_ until D_31_ detected with Mini-FLOTAC technique.

For *T*. *trichiura*, the observed prevalence remained constantly 100% until the last detection (98%), for *A*. *lumbricoides* it was always above 77%, and for hookworm ranged between 63% and 84% (Table 2).

### Microscopic Readability

Over the course of our study, the shape and unique identification of hookworm eggs gradually deteriorated, and hence, hookworm eggs became progressively less readable. Meanwhile, eggs of *A*. *lumbricoides* and *T*. *trichiura* were clearly visible and readable at the same ease throughout the study.

**Fig 4:**
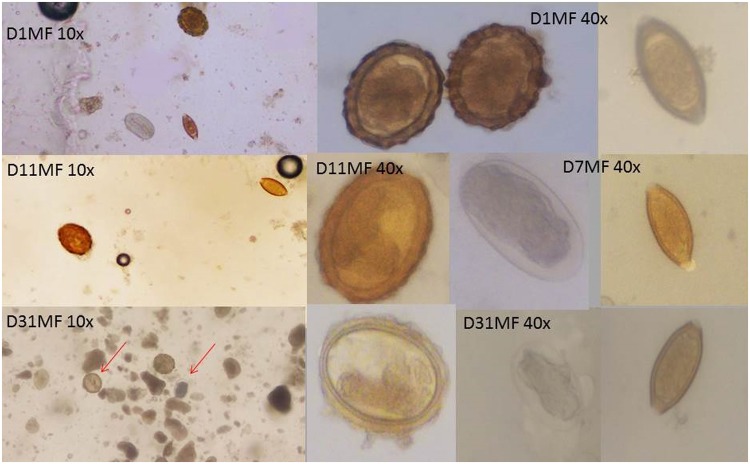
Pictures of soil-transmitted helminths at different time points after fixation in formalin. D_1_ 10x: *A*. *lumbricoides*, *T*. *trichiura*, and hookworm; D_1_ 40x: *A*. *lumbricoides* and *T*. *trichiura*; D_7_ 40x hookworm and *T*. *trichiura*; D_11_ 10x *A*. *lumbricoides* and *T*. *trichiura*; D_11_ 40x *A*. *lumbricoides*, D_31_ 10x *A*. *lumbricoides* and hookworm, D_31_ 40x *A*. *lumbricoides*, hookworm, and *T*. *trichiura*.

Sequential microphotographs are presented in Fig 4. The external membrane of the hookworm eggs started to fade and be less recognizable from D_11_ onwards, hence 12 days after stool production and fixation in 5% formalin. It became increasingly difficult to read hookworm eggs until the end of the study (D_31_) when the eggs were very hard to recognize. Progressive degradation was not observed for the other soil-transmitted helminth eggs, as shown in Fig 4. The *A*. *lumbricoides* eggs sometime became decorticated and *T*. *trichiura* eggs showed larvae inside, but for both these two species the eggs were still perfectly recognizable at the final observation time point at D_31_.

## Discussion

The possibility to fix and examine fecal samples after days and weeks from collection is an important feature to facilitate the integration of soil-transmitted helminthiasis surveys with other NTDs, such as lymphatic filariasis and trachoma [13, 14, 16, 28, 29]. Only few studies, however, have been conducted on preserved stool samples [16, 30–32], especially on the effect of preservation of samples over time either on eggs detection or fecal egg counts. Therefore the time limit, beyond which qualitative and quantitative diagnosis for soil-transmitted helminth infections becomes unreliable, needs to be examined. In the current study, we assessed the effect of 5% formalin (ratio 1:1) preservation on helminth eggs diagnostic accuracy over a 31-day time period using Mini-FLOTAC. The 1:1 dilution has been used before in studies on comparison among different copromicroscopic techniques [17, 32, 33], but never as a preservative concentration; usually the storage was carried out with 10% or 5% formalin at 1:4 dilution. The performance of Mini-FLOTAC was also compared with Kato-Katz, using fresh stool samples. Of note, Kato-Katz is the WHO-recommended diagnostic method for soil-transmitted helminth infection (and intestinal schistosomiasis) [26] and is indeed widely used [11].

Our study provides new insight into the timing of preservation for an accurate estimate of prevalence and intensity of soil-transmitted helminth infections over the course of stool preservation. Importantly, prevalence estimate for each of the three soil-transmitted helminth species did not change significantly over the 31-day observation period. For *T*. *trichiura*, the observed prevalence remained 100% throughout. With regard to *A*. *lumbricoides*, the prevalence did not vary greatly over the examination period. The greatest variation was noted for hookworm: over the first 8 days the steepest drop of prevalence (from 66% to 51%) was observed, which then remained stable at 51% until the final day of analysis (D_31_). The Pearson index of correlation resulted to be stable throughout the study, which suggests a good effect on preservation and accordance among detections, and only slight changes in the prevalence and intensity for each observation during the 31 days of the study. The major change in prevalence was linked to low-intensity infections. *T*. *trichiura* and *A*. *lumbricoides* were mainly of light and moderate infection intensities and only two children had heavy *A*. *lumbricoides* infection. With regard to hookworm, all infections were of light intensity. Furthermore, since many children did have very low infections approaching 10 or lower EPGs, being so close to Mini-FLOTAC detection limit, it considerably affected the variation of prevalence estimates. The mean fecal egg counts of hookworm infections remained stable until 2 weeks from collection, whilst for *T*. *trichiura* and *A*. *lumbricoides* it did not change throughout the 31-day observation period. As shown in Fig 3 the classes of intensity of infection were consistent throughout the study for all the children.

Although the main aim of this study was not to compare the performance of Mini-FLOTAC and Kato-Katz technique, which had been previously assessed in a larger studies [32, 33], we tested fresh samples with both techniques at baseline to validate the prevalence data and to confirm whether the accuracy of the two techniques was comparable. In fact, we noted that Mini-FLOTAC technique resulted to be as sensitive as a single Kato-Katz thick smear with no statistical differences among detections for the three soil-transmitted helminth species. There was a variation in sensitivity for hookworm among Mini-FLOTAC detections and this could be explained by the aforementioned limit of the current study, as for hookworm many infections were light and close to the sensitivity threshold. It is to be noted that the flotation solution used in this study is not the most suitable to detect *A*. *lumbricoides*. As reported from other studies [32] the most appropriate flotation solution to for the diagnosis of *A*. *lumbricoides* is zinc sulphate; but even if the sensitivity of the latter was higher compared to the flotation solution no. 2, the mean fecal egg counts were lower [33]. Moreover, the zinc sulphate solution is more expensive and less easy to supply in low-resource setting.

In conclusion, the findings of our study suggest that for a qualitative diagnosis with Mini-FLOTAC, stool samples fixed with 5% formalin can be preserved at least one month without impairing the quality of the data on prevalence of soil-transmitted helminth infections. However, for an accurate quantitative diagnosis for hookworm, we suggest a maximum of 15 days of preservation; after this time, hookworm eggs start to deteriorate and the consistence of microscope reading decreases (unless the reader places additional attention to detect hookworm eggs), and the fecal egg counts progressively decline. As for *A*. *lumbricoides* and *T*. *trichiura*, eggs remain stable over one month and therefore a longer preservation might still give accurate data on intensity of infections. Further studies are needed to explore the performance of stool preserved with formalin at different concentrations and dilutions, or with other preservatives, and possibly these studies should be carried out in areas where hookworm infections are moderate and/or heavy. Additionally, studies should determine the effect longer fixation periods (perhaps up to 2 or 3 months of stool preservation in formalin) to evaluate the durability of *A*. *lumbricoides* and *T*. *trichiura* eggs. Data from this study are pivotal for the use of Mini-FLOTAC as an alternative to Kato-Katz, which would allow the integration of soil-transmitted helminthiasis into TAS surveys, and hence, integrated monitoring and evaluation of lymphatic filariasis with soil-transmitted helminthiasis, as recommended recently by WHO [34].
